# Auditor Choice and the Informativeness of 10-K Reports

**DOI:** 10.1177/0148558X211062430

**Published:** 2021-12-15

**Authors:** Karel Hrazdil, Dan A. Simunic, Nattavut Suwanyangyuan

**Affiliations:** 1 Simon Fraser University, Burnaby, British Columbia, Canada; 2 Brock University, St. Catharines, Ontario, Canada

**Keywords:** auditor competencies, Big 4 auditors, 10-K disclosure volume, audit quality

## Abstract

This study provides new evidence on the influential role of external auditors in enhancing the informativeness of form 10-K annual reports to shareholders. Specifically, we find that the client’s choice of a Big 4 auditor (PwC, EY, KPMG, and Deloitte) versus a non-Big 4 auditor contributes to cross-sectional variations in 10-K disclosure volume. We also document that the benefit of enhanced disclosures provided by Big 4 auditors is more pronounced for audit clients with poorer accrual quality and those with higher information asymmetry. Furthermore, we introduce the portion of 10-K length unexplained by operating complexity and observable client characteristics as a new proxy for audit firm effort. Specifically, we find that abnormally long disclosures are associated with higher audit fees and longer audit report lag, which implies that an incremental level of audit effort can be inferred from the discretionary component of 10-K disclosures. As audit effort is costly, a greater volume of 10-K disclosures can be expected to be associated with an improvement in the quality of financial reporting. Overall, our findings show that auditors play more than a simple attestation role in the financial reporting process, and that the quality of financial reporting in a company’s 10-K annual report is a joint product of the effort and decisions of both a company’s managers and its auditors.

## Introduction

While the standard audit report clearly states that an auditor’s responsibility is to express an opinion on financial statements, there is controversy over whether the role of the external auditor is limited to simply verifying compliance with generally accepted accounting principles (GAAP) or whether that responsibility extends to assuring “fair presentation” to the capital markets (e.g., DeFond et al., 2016). This debate occurs because, while *management* is clearly responsible for the preparation and fair presentation of financial statements that are free from material misstatement, the *auditor* must obtain reasonable assurance that this is indeed the case. An auditor does this by planning and performing the audit to obtain sufficient, appropriate audit evidence that financial information is fairly presented, which includes assessment of the accounting principles used and of the significant accounting estimates made by management, as well as an evaluation of the overall financial statement presentation. Therefore, it is an empirical question whether the choice of auditor leads to significant variations in disclosures in 10-K annual reports.

Auditors can influence 10-K disclosures through two basic channels. First, a significant part of the financial statements included in a form 10-K consists of narrative footnote disclosures, and an auditor is considered an “expert” with respect to such disclosures. That is—as with financial statement amounts—the auditor must obtain reasonable assurance concerning the fair presentation of disclosures in footnotes. Second, contrary to popular belief about the role of the auditor, auditing standards (AS 2710: Other Information in Documents Containing Audited Financial Statements) explicitly require auditors to read the entire annual report and consider whether unaudited information, for example, Management Discussion & Analysis, or the manner of its presentation, is materially inconsistent with the information provided, or the manner of its presentation, which appears in the audited financial statements American Institute of Certified Public Accountants (AICPA), 1997. This requirement is consistent with a detailed summary of observations from roundtable discussions^1^ on the evolving role of the auditor, which states,

Less sophisticated investors may not be aware that auditors currently provide some value by reading other information provided outside of the audited financial statements for consistency with the audited financial statements. (Center for Audit Quality, 2011, p. 7)

In this study, we focus on determining whether a client’s choice of a Big 4 auditor contributes to cross-sectional variations in 10-K informativeness as measured by disclosure volume. This approach is consistent with Dunn and Mayhew (2004), who argue that a client’s choice of an industry specialist auditor is associated with the client’s intention to provide enhanced disclosure. However, instead of the now-discontinued AIMR scores used by those authors, we use the length of 10-K reports (e.g., Li, 2008; Loughran & McDonald, 2014) over the 11-year period from 2004 through 2014, and find that the choice of a Big 4 auditor is positively associated with 10-K disclosure volume in both the full sample and the propensity score matching (hereafter PSM) sample (e.g., Lawrence et al., 2011). This result is consistent with product differentiation between Big 4 and non-Big 4 auditors, and that such differentiation is associated with variations in 10-K informativeness, as measured by disclosure volume.

There is strong evidence that Big 4 auditors provide higher quality attestation (i.e., a higher level of assurance) than do non-Big 4 auditors, and the former are associated with either a lower level of discretionary accruals (e.g., Becker et al., 1998; J. R. Francis et al., 1999) or a reduction in information asymmetry (e.g., Jensen & Meckling, 1976; Watts & Zimmerman, 1983). We therefore expect the association between Big 4 auditor choice and 10-K disclosure volume to be stronger in situations where users of financial reports potentially need more information to understand the effects of material transactions and/or events. Consistent with our expectations, we find that the benefit of enhanced disclosures provided by Big 4 auditors is more pronounced for audit clients with poorer accrual quality and for those with higher information asymmetry, supporting the notion that the choice of a Big 4 auditor signals a client’s intention to provide not only more assurance but also enhanced disclosure quality (e.g., Baginski et al., 2004; D’Souza et al., 2010; Hutton et al., 2003; Mercer, 2004).

Finally, we provide new evidence that the portion of 10-K length unexplained by operating complexity and observable client characteristics induces higher audit effort and is associated with higher audit fees and longer audit report lags. In other words, abnormally long disclosures are consistent with external auditors exerting greater effort, charging an audit fee premium (Simunic & Stein, 1996), and experiencing an increase in audit report lag (Knechel & Payne, 2001).

Our study makes several contributions to the literature. First, the findings help to answer the broader question of how auditor choice is associated with the quality of a firm’s disclosure by providing evidence that the choice of a Big 4 auditor is associated with enhanced disclosure practices in 10-K reports. This suggests that the auditor’s role in financial reporting is not limited to simply providing assurance concerning management’s compliance with GAAP. Second, this study fills a gap in the literature regarding the textual analysis of corporate disclosures, as prior studies have mainly focused on the managerial discretion in firms’ disclosure practices. In contrast, we highlight the extent to which a client’s choice of a Big 4 auditor contributes to variations in disclosure practices in 10-K reports. Given the regulatory concerns regarding corporate disclosure and the trend toward more detailed disclosure, this study provides useful insights into the evolving role of external auditors in the reporting process and should be of interest to both the Securities and Exchange Commission (SEC) and the Public Company Accounting Oversight Board (PCAOB). Finally, because an abnormally long disclosure likely requires additional audit effort, we contribute to the literature by demonstrating that overall auditor effort can be inferred from a discretionary component of 10-K disclosure volume.

The remainder of this article is organized as follows. The “Literature Review and Hypotheses Development” section reviews the relevant literature and develops the research hypotheses. Next, we present our research design. We report sample characteristics and descriptive statistics in the “Sample Selection and Descriptive Statistics” section. The “Main Results” section presents our empirical results and additional analyses for robustness checks. Finally, we offer several conclusions to the study.

## Literature Review and Hypotheses Development

This study builds on and contributes to two areas of research: (a) research on audit firm product differentiation and differential audit quality, and (b) research on corporate reporting and disclosure.

### Research on Audit Firm Product Differentiation and Differential Audit Quality

Simunic and Stein (1987) argued that the output of the audit service is likely to be multidimensional. That is, audit services likely possess multiple characteristics that may be valued by purchasers, namely, the top managers of audited entities. This view is consistent with Lancaster (1966) who argued that goods and services in general can be described by an implicit vector of valued characteristics and an implicit vector of prices per unit of each characteristic, with the observed market price being the inner product of these vectors. For example, automobiles can be thought of as containing various characteristics, such as *transportation, style, safety*, and *driving entertainment*, and each characteristic has an implicit price, which when taken together determine the price of a car. Simunic and Stein (1987) posited that external audits contain three characteristics, termed *internal control, credibility*, and *product line*. They then focused on and examined the implications of differences in *credibility* on auditor choice in the Initial public offering (IPO) market.

Subsequent research has largely viewed an audit as being one-dimensional and has focused on the attribute of *assurance (credibility)* and systematic differences in assurance levels, which are equated with differences in audit quality. There is no consensus as to the best measure of audit quality, because different perspectives on audit quality imply different proxies to measure it (e.g., PCAOB, 2015). One of the most-cited definitions of audit quality is from DeAngelo (1981), who states that “the quality of audit services is defined to be the market-assessed joint probability that a given auditor will both discover a breach in the client’s accounting system and report the breach” (p. 186). More importantly, DeAngelo argues that large auditors are expected to have stronger incentives and competencies to supply high-audit quality, which has motivated much of the auditing literature to use auditor size as a proxy for audit quality. Existing research provides ample evidence that Big 4 auditors deliver higher audit quality, as captured by various output-based audit quality proxies, including a lower incidence of accounting fraud (e.g., Lennox & Pittman, 2010), a lower incidence of accounting restatements (e.g., Eshleman & Guo, 2014), lower discretionary accruals (e.g., Becker et al., 1998; J. R. Francis et al., 1999), higher audit fees (e.g., Craswell et al., 1995; Hay et al., 2006), increased Earnings response coefficients (ERCs) (e.g., Teoh & Wong, 1993), improved analyst earnings forecasts (e.g., Behn et al., 2008), and a lower cost of debt and equity (e.g., Khurana & Raman, 2004).

The auditing literature also provides compelling evidence that the client’s choice of auditor potentially signals client incentives to demand high-audit quality, as evidenced by the stock market’s reaction to auditor switches (e.g., Boone & Raman, 2001; Chang et al., 2010; Khalil et al., 2011; Knechel et al., 2007) and enhanced disclosure quality (Dunn & Mayhew, 2004). This evidence is consistent with the survey results of the Government Accountability Office (2008), which indicate that the ability to handle complex company operations, technical capabilities, and industry expertise are considered major reasons why large public companies primarily choose Big 4 audit firms as their external auditors. More recently, a number of studies have questioned whether Big 4 auditors provide higher audit quality in the current auditing environment than do non-Big 4 auditors (e.g., DeFond et al., 2017; Eshleman & Guo, 2014; J. Jiang et al., 2019; Lawrence et al., 2011), and they have highlighted the need for more evidence on the Big 4 effect.^2^

While a systematic difference between Big 4 and non-Big 4 audit quality, as measured by proxies for assurance levels, is well documented in the literature, we argue that audit services may also contain a second valued characteristic that can be termed *financial reporting quality*. As a result, a Big 4 audit can differ from a non-Big 4 audit both in terms of the *assurance* level provided and the level of *financial reporting quality* provided. Whether Big 4 audits provide a higher, equal, or lower level of *financial reporting quality* than non-Big 4 audits is unknown, as any of these relationships could conceivably exist.^3^ However, we consider it likely that, if there is a difference between Big 4 and non-Big 4 audits on this characteristic, the relationship is positively correlated.

### Research on Corporate Reporting and Disclosure

A large body of research on corporate reporting and disclosure has focused on the benefits of increased disclosures and has argued that an increased volume of firm disclosures (both narrative and numerical) is associated with reduced information asymmetry, higher trading activity, and an overall improvement in the efficiency of information price discovery (e.g., Balakrishnan et al., 2014; Botosan, 1997; Diamond & Verrecchia, 1991; Graham et al., 2005; Leuz & Verrecchia, 2000). Consistent with the empirical evidence on the beneficial role of detailed corporate disclosure in a global setting (Lang & Stice-Lawrence, 2015), Chung et al. (2019) find that both textual quantity and numerical quantity are associated with an overall improvement in the efficiency of information price discovery for a large sample of companies traded on major U.S. stock exchanges.

However, there is also a line of research on the textual analysis of corporate disclosures that raises significant concerns regarding the relevance of information in financial disclosures based on the presumption that “longer and less readable documents are more deterring and require higher costs of information-processing” (Li, 2008, p. 222). For example, Nelson and Pritchard (2007) find that companies that are subject to more shareholder litigation use more readable language in their reports and avoid boilerplate warnings, while You and Zhang (2009) document that investors underreact to the information provided in 10-K filings, with a more pronounced effect for companies that file more complex and less readable 10-K reports. Lawrence (2013) also finds that individual investors are more likely to invest in firms that provide clear and concise disclosures relative to other firms. While these effects may sometimes exist, in the context of financial statement footnotes, which form a large part of 10-K disclosures and where auditors are considered to be “experts,” it is more difficult to argue that greater length inhibits investor understanding. For example, short “boilerplate” disclosures of contingent liabilities associated with ongoing litigation are certainly less informative than a clear, detailed description of litigation and its possible consequences to a company. Together, these findings suggest that detailed and lengthy disclosures may potentially create a risk of information overload and make it more difficult for the intended users to identify the information that is most relevant, but this is less a concern in the area (i.e., footnote disclosures) where the auditor has the greatest responsibility.

The 10-K disclosure volume, as measured by the number of words in a 10-K filing, was first introduced to capture annual report readability (e.g., Li, 2008; Loughran & McDonald, 2014). In a subsequent study by Loughran and McDonald (2016), the authors argue that it is not possible to disentangle the complexity of a firm’s business from the readability of its annual reports, and they recommend that researchers focus on a broader concept of information complexity. Cazier and Pfeiffer (2015) use a small sample of 10-Ks and partition the disclosure volume of 10-K reports into three major components: (a) firms’ operating complexity, (b) disclosure redundancy, and (c) residual disclosure. The authors argue that while the disclosure volume of 10-K reports is largely driven by operating complexity and disclosure redundancies, a substantial amount of disclosure volume is attributable to a discretionary reporting choice by management; hence, they call for future research to investigate the factors that drive idiosyncratic disclosure, which is not explained by either operating complexity or disclosure redundancies. More recently, Cazier and Pfeiffer (2017) find evidence that repetition of information in 10-K reports is a strategic response to managers’ reporting incentives to obfuscate relevant information when firm performance is poor and to highlight favorable news when firms are performing well.

Prior studies further indicate that the choice of external auditor has a significant influence on client disclosure quality. For example, Dunn and Mayhew (2004) show that industry specialists provide value-added services, including disclosure advice, to their audit clients in the form of improved disclosure quality. This is because, when determining whether financial statements are fairly presented or not, an auditor has to consider whether the “information presented in the financial statements, including accounting policies, is relevant, reliable, comparable, and understandable,” and whether the “financial statements provide sufficient disclosures to enable users to understand the effect of material transactions and events on the information conveyed in the financial statements” (PCAOB, 2005, p. 8). Thus, the active role of the external auditor in the client’s accounting and disclosure choices affects the content of the client’s financial statements because the auditor must ensure that the financial statements are appropriate (Gibbins et al., 2001).

### Hypotheses Development

The argument leading to our first hypothesis is based on the notion that audit services possess two valued characteristics, *assurance level* and *financial reporting quality*, and that the Big 4 firms are quality differentiated from the non-Big 4 firms on both dimensions. This is consistent with the broader view of auditors’ responsibilities raised by DeFond et al. (2016). Although both Big 4 and non-Big 4 auditors are held to the same regulatory and professional standards, financial statement users expect high-quality auditors to consider more than technical GAAP compliance when determining whether financial statements are fairly presented. Therefore, whether and to what extent the choice of Big 4 auditor affects *financial reporting quality* as measured by the disclosure volume of 10-K reports is an empirical issue and the focus of this study.

Because theory suggests that Big 4 auditors have greater incentives to maintain high levels of audit quality overall, and that Big 4 auditors are utilized because of their incentives and competencies to enhance the credibility of financial reporting,^4^ the choice of Big 4 auditors should therefore help audit clients improve the informativeness of their disclosures. Instead of using the now-discontinued AIMR scores, as in Dunn and Mayhew (2004), we use 10-K disclosure volume to capture the informativeness of client disclosures.^5^

The existing literature often uses text-based analyses to estimate various proxies for readability and complexity, such as 10-K document length and file size, based on the general consensus that firms with annual reports that are less complex and easier to read have more persistent positive earnings, experience smaller underreactions to earnings news, and attract more individual investors (Lawrence, 2013; You & Zhang, 2009). Part of the evidence can be attributed to Bloomfield’s (2002) Incomplete Revelation Hypothesis, which states that statistics that are more costly to extract from public data are less completely revealed in market prices. Thus, because less readable and more complex 10-K reports likely provide managers with more opportunities to withhold bad news from the market, this line of reasoning hypothesizes a negative association between the choice of Big 4 auditors and 10-K disclosure volume, which would indicate that the clients of Big 4 auditors benefit from clear and concise corporate disclosures.

However, as Bloomfield (2008) later notes, firms could simply require longer and more detailed explanations to support certain complex structural transactions and events. They could respond to changes in their information environment by voluntarily increasing both the quantity and frequency of their filings relative to what is mandated by market regulators. If more detailed annual reports reflect new value-relevant information and are indicative of higher reporting quality, this line of reasoning implies a positive association between the choice of Big 4 auditors and 10-K disclosure volume, which would indicate that the clients of Big 4 auditors benefit from improved disclosure quality through longer and more detailed disclosures.

In sum, whether and to what extent the choice of Big 4 auditors affects the disclosure volume of 10-K reports is an empirical issue. The first hypothesis is therefore formulated, in null form, as follows:

**Hypothesis 1:** The choice of a Big 4 auditor is not associated with 10-K disclosure volume.

Next, we investigate to what extent the choice of a Big 4 auditor impacts 10-K disclosure volume in the two following situations where financial reporting users potentially need more information to understand the effects of material transactions and events on the information conveyed in the financial disclosure. First, while prior research provides evidence that the clients of Big 4 auditors report lower discretionary accruals on average than those of non-Big 4 auditors (e.g., Becker et al., 1998; J. R. Francis et al., 1999), we hypothesize that the influence of Big 4 auditors on 10-K disclosure volume will be more pronounced for clients with poorer accrual quality, as measured by the magnitude of discretionary accruals, thus supporting these clients’ attempts to increase the credibility of their financial reports. Second, because companies seek to shape their information environment by voluntarily disclosing more information to reduce information asymmetries (e.g., Balakrishnan et al., 2014), we use the effective bid–ask spread as a proxy for information asymmetry, and we hypothesize that the influence of Big 4 auditors on 10-K disclosure volume will be more pronounced for clients with higher levels of information asymmetry, thus supporting these clients’ attempts to decrease the information asymmetries. Taken together, the second set of hypotheses is formulated as follows:

**Hypothesis 2a:** The association between the magnitude of discretionary accruals and 10-K disclosure volume increases with the presence of a Big 4 auditor.
**Hypothesis 2b:** The association between the effective bid–ask spread and 10-K disclosure volume increases with the presence of a Big 4 auditor.

Finally, the production of a higher level of *financial reporting quality* is costly, and we expect that more detailed financial reporting requires higher costs for information processing (e.g., Bloomfield, 2002; Li, 2008) along with more effort in performing the audit service. Given that the auditor’s effort level is not observable, researchers often use audit hours to measure audit effort (e.g., Caramanis & Lennox, 2008; Cho et al., 2017; Palmrose, 1986). However, because of the limited availability of audit hours, audit effort can also be inferred from a variety of observable auditor responses, such as audit fees (Albring et al., 2018; Simunic & Stein, 1996) and audit report lags (Amin et al., 2018; Knechel & Payne, 2001). This is partly because auditors can reduce the risk of undetected material misstatement by increasing their effort in response to increased inherent and control risks (W. Jiang & Son, 2015) and litigation risks (McCracken, 2002) due to undetected material misstatements, which is reflected in increased audit fees and/or the time required to complete audits. Therefore, if *financial reporting quality* is a valued characteristic of an audit, and the use of a Big 4 versus non-Big 4 audit firm is associated with variations in 10-K disclosure volume, we predict that the residual disclosure of 10-K reports will be associated with either higher audit fees or longer audit report lags. In other words, incremental audit effort, as measured by the amount by which *actual* 10-K disclosure volume exceeds *predicted* 10-K disclosure volume, would reflect a greater audit effort in providing assurance services to audit clients. The third set of hypotheses is formulated, in null form, as follows:

**Hypothesis 3a:** Higher residual disclosure is not associated with higher audit fees.
**Hypothesis 3b:** Higher residual disclosure is not associated with longer audit report lags.

## Research Design

To test the first hypothesis, we include an indicator variable for Big 4 audit firms to examine the differential effect of Big 4 auditor choice on 10-K disclosure volume. Based on existing disclosure studies (e.g., Cazier & Pfeiffer, 2015; Li, 2008), we estimate the following empirical model (Equation 1: the disclosure model); definitions of the variables are presented in Table 1.

(1) $$\;\begin{array}{c} \mathrm{LNWORD}S_{t}=\alpha_{0}+\alpha_{1}\mathrm{BIG}4_{t}+\alpha_{2}\mathrm{DELTA}\_\_\mathrm{RO}A_{t}+\alpha_{3}\mathrm{DELTA}\_\_\mathrm{RE}V_{t}\\ +\alpha_{4}MA_{t}+\alpha_{5}\mathrm{FYRE}T_{t}+\alpha_{6}\mathrm{SD}\_\_\mathrm{RETUR}N_{t}+\alpha_{7}\mathrm{SPI}\_\_DM_{t}\\ +\alpha_{8}\mathrm{CAP}\_\_\mathrm{LEAS}E_{t}+\alpha_{9}\mathrm{OP}\_\_\mathrm{LEAS}E_{t}+\alpha_{10}RD_{t}+\alpha_{11}I\mathrm{NTAN}G_{t}\\ +\alpha_{12}\mathrm{SIZ}E_{t}+\alpha_{13}\mathrm{AG}E_{t}+\alpha_{14}\mathrm{MT}B_{t}+\alpha_{15}\mathrm{LEVERAG}E_{t}\\ +\alpha_{16}\mathrm{FC}F_{t}+\alpha_{17}\mathrm{DERIVATIV}E_{t}+\alpha_{18}\mathrm{LNBUSSE}G_{t}\\ +\alpha_{19}\mathrm{LNGEOSE}G_{t}+\alpha_{20}\mathrm{SD}\_\_\mathrm{OIAD}P_{t}+\alpha_{21}\mathrm{DELAWAR}E_{t}\\ +\alpha_{22}\mathrm{IP}O_{t}+\alpha_{23}\mathrm{SE}O_{t}+\alpha_{24}\mathrm{NMCOUN}T_{t}\\ +\mathrm{Year}\;\mathrm{and}\;\mathrm{Industry}\;\mathrm{Fixed}\;\mathrm{Effects}+\varepsilon_{t}. \end{array}$$

**Table 1.** Descriptive Statistics—The Disclosure Model.

Panel A: The Full Sample.						
	Non-Big 4 firms (*n* = 13,818)		Big 4 firms (*n* = 29,757)		Differences in means	
Variables	*M*	*SD*	*M*	*SD*	Differences	*t*-statistic
Disclosure attributes						
*LNWORDS*	10.45	0.47	10.80	0.46	–0.35	–72.13***
Client characteristics						
*LNASSET*	5.08	1.61	7.16	1.62	–2.08	–113.33***
*ATURN*	0.89	0.94	0.96	0.93	–0.07	–7.17***
*CURRENT*	3.35	5.18	3.22	5.24	0.13	2.49**
*LEVERAGE*	0.15	0.20	0.22	0.19	–0.06	–32.98***
*ROA*	–0.02	0.19	0.02	0.18	–0.04	–20.23***
Determinants of disclosure attributes						
*DELTA_ROA*	–0.31	3.38	–0.24	3.61	–0.06	–1.75*
*DELTA_REV*	0.17	0.47	0.13	0.51	0.04	7.42***
*MA*	0.11	0.36	0.22	0.37	–0.11	–29.58***
*FY_RET*	–0.13	0.77	–0.11	0.75	–0.03	–3.48***
*SD_RETURN*	0.13	0.07	0.11	0.07	0.02	26.01***
*SPI_DM*	0.46	0.50	0.68	0.50	–0.23	–45.05***
*CAP_LEASE*	0.43	0.50	0.53	0.50	–0.10	–19.04***
*OP_LEASE*	0.37	0.49	0.41	0.49	–0.04	–8.56***
*RD*	0.04	0.11	0.04	0.10	0.00	–3.69***
*INTANG*	0.12	0.23	0.18	0.22	–0.06	–27.79***
*SIZE*	4.46	1.28	6.96	1.29	–2.50	–161.30***
*AGE*	2.47	0.82	2.67	0.80	–0.20	–22.88***
*MTB*	1.78	1.58	1.85	1.61	–0.07	–4.29***
*FCF*	0.02	0.14	0.07	0.15	–0.05	–32.84***
*DERIVATIVE*	0.11	0.35	0.28	0.35	–0.17	–47.23***
*LNBUSSEG*	0.70	0.54	0.96	0.54	–0.26	–44.55***
*LNGEOSEG*	0.59	0.68	0.88	0.67	–0.30	–43.75***
*SD_OIADP*	0.12	0.25	0.08	0.28	0.04	15.76***
*DELAWARE*	0.41	0.50	0.61	0.50	–0.20	–39.30***
*IPO*	0.00	0.04	0.00	0.03	0.00	3.15***
*SEO*	0.07	0.28	0.09	0.28	–0.03	–10.48***
*NMCOUNT*	5.62	0.20	5.74	0.22	–0.11	–49.80***
Other tested variables						
*ADA_MJR*	0.07	0.07	0.05	0.06	0.03	30.42***
*EFFSPRD*	1.15	0.82	0.29	0.79	0.86	72.22***
Panel B: The PSM Sample.						
	Non-Big 4 firms (*n* = 6,576)		Big 4 firms (*n* = 6,576)		Differences in means	
Variables	*M*	*SD*	*M*	*SD*	Differences	*t*-statistic
Disclosure attributes						
*LNWORDS*	10.57	0.47	10.61	0.46	–0.04	–5.51***
Client characteristics						
*LNASSET*	5.57	1.61	5.58	1.63	–0.02	–0.64
*ATURN*	0.97	0.94	0.95	0.92	0.02	1.31
*CURRENT*	3.64	5.24	3.59	5.19	0.05	0.56
*LEVERAGE*	0.17	0.20	0.17	0.19	0.00	0.03
*ROA*	–0.01	0.19	–0.02	0.18	0.00	1.01
Determinants of disclosure attributes						
*DELTA_ROA*	–0.29	3.38	–0.31	3.61	0.01	0.21
*DELTA_REV*	0.17	0.47	0.17	0.51	0.00	0.04
*MA*	0.15	0.36	0.16	0.37	–0.01	–0.94
*FY_RET*	–0.14	0.77	–0.15	0.75	0.01	0.69
*SD_RETURN*	0.12	0.07	0.12	0.07	0.00	–0.04
*SPI_DM*	0.55	0.50	0.55	0.50	0.00	–0.16
*CAP_LEASE*	0.50	0.50	0.51	0.50	–0.01	–0.66
*OP_LEASE*	0.42	0.49	0.42	0.49	–0.01	–0.60
*RD*	0.05	0.11	0.05	0.10	0.00	0.03
*INTANG*	0.14	0.23	0.14	0.22	0.00	–0.89
*SIZE*	5.25	1.28	5.24	1.29	0.01	0.47
*AGE*	2.50	0.82	2.49	0.80	0.01	0.81
*MTB*	1.88	1.58	1.85	1.61	0.03	1.01
*FCF*	0.04	0.14	0.03	0.15	0.00	1.31
*DERIVATIVE*	0.15	0.35	0.14	0.35	0.00	0.27
*LNBUSSEG*	0.82	0.54	0.82	0.54	0.00	0.47
*LNGEOSEG*	0.73	0.68	0.73	0.67	0.00	0.18
*SD_OIADP*	0.11	0.25	0.11	0.28	0.00	–0.03
*DELAWARE*	0.53	0.50	0.53	0.50	0.00	–0.05
*IPO*	0.00	0.04	0.00	0.03	0.00	0.77
*SEO*	0.09	0.28	0.08	0.28	0.00	0.59
*NMCOUNT*	5.68	0.20	5.68	0.22	0.00	0.36
Other tested variables						
*ADA_MJR*	0.06	0.07	0.06	0.06	0.01	5.29***
*EFFSPRD*	0.72	0.87	0.69	0.79	0.03	1.77*

*Note.* This table reports the summary statistics of variables used in the full sample (Panel A) and the PSM samples (Panel B) from fiscal year 2004 to 2014. The variables are defined as follows—Disclosure attributes: *LNWORDS* = the natural logarithm of the word count in the 10-K complete submission text file; *LNASSET* = the natural logarithm of total assets (in millions) at the end of the fiscal year; *ATURN* = the ratio of sales to lagged total assets; *CURRENT* = the ratio of current assets to current liabilities; *LEVERAGE* = the sum of short-term and long-term debt in year *t*, divided by total assets; *ROA* = income before extraordinary items, scaled by average total assets. Determinants of disclosure attributes: *DELTA_ROA* = the annual change in ROA; *DELTA_REV* = the annual percentage change in sales; *MA* = indicator variable equal to one if an audit’s client is engaged in a merger or acquisition during the year, and zero otherwise; *FY_RET* = raw annual return over the 12-month fiscal period; *SD_RETURN* = the standard deviation of the monthly stock returns in the prior fiscal year; *SPI_DM* = indicator variable equal to one if an audit’s client has any special item during the year, and zero otherwise; *CAP_LEASE* = indicator variable equal to one if the company reports a capital lease on its balance sheet, and zero otherwise; *OP_LEASE* = indicator variable equal to one if the value of operating lease payments due in 1 year is greater than 1% of total assets, and zero otherwise; *RD* = the amount of research and development expense, scaled by lagged assets; *INTANG* = the unamortized value of purchased intangible assets, scaled by lagged assets; *SIZE* = the natural logarithm of the firm’s market value at the end of the fiscal year; *AGE* = the natural logarithm of the number of years since a firm’s first appearance in the Compustat annual files; *MTB* = the firm’s market value divided by its book value; *SPECIAL* = the amount of special items, scaled by total assets; *FCF* = the average operating cash flows scaled by total assets over the current and prior years; *DERIVATIVE* = indicator variable equal to one if the company reports any current or accumulated gains or losses on derivative transactions, and zero otherwise; *LNBUSSEG* = the natural logarithm of one plus the number of business segments; *LNGEOSEG* = the natural logarithm of one plus the number of geographic segments; *SD_OIADP* = the standard deviation of the operating earnings in the last 5 fiscal years; *DELAWARE* = indicator variable equal to one if an audit’s client is incorporated in Delaware, and zero otherwise; *IPO* = indicator variable equal to one if an audit’s client is engaged in an initial public offering during the year, and zero otherwise; *SEO* = indicator variable equal to one if an audit’s client is engaged in any seasoned equity offering during the year, and zero otherwise; *NMCOUNT* = the natural logarithm of the number of non-missing items in Compustat annual files. Other tested variables: *BIG4* = indicator variable equal to one if the firm’s auditor is a member of the Big 4 audit firms (PwC, EY, KPMG, and Deloitte), and zero otherwise; *ADA_MJR* = the absolute value of discretionary accruals based on the modified Jones model (Dechow et al., 1995) controlling for firm’s financial performance (Kothari et al., 2005); *EFFSPRD* = the difference between the bid–ask midpoint and the actual transaction price divided by the bid–ask midpoint, following the same approach as in Hendershott et al. (2011). All continuous variables are winsorized at the 1st and 99th percentiles. PSM = propensity score matching.

* , **, and *** denote significance at the .10, .05, and .01 levels, respectively, using two-tailed *t* tests of differences in means.

To address the identification concerns related to functional form misspecification (e.g., Boone et al., 2010; Lawrence et al., 2011),^6^ we use a PSM model to control for differences in client characteristics between Big 4 and non-Big 4 auditors while estimating auditor treatment effects.^7^ Specifically, we estimate the following logistic regression (Equation 2: the auditor selection model) and obtain the probability of hiring a Big 4 auditor based on a broad range of observable client characteristics, including asset size, asset turnover, current ratio, financial leverage, and firm performance, together with the control variables used in the disclosure model.

(2) $$\begin{array}{c} \mathrm{BIG}4_{t}=\alpha_{0}+\alpha_{1}\mathrm{LNASSE}T_{t}+\alpha_{2}\mathrm{ATUR}N_{t}+\alpha_{3}\mathrm{CURREN}T_{t}+\alpha_{4}\mathrm{LEVERAG}E_{t}\\ \;\;\;\;+\alpha_{5}\mathrm{RO}A_{t}+\Sigma \mathrm{Control}\;\mathrm{variables}+\mathrm{Year}\;\mathrm{and}\;\mathrm{Industry}\;\mathrm{Fixed}\;\mathrm{Effects}+\varepsilon_{t}. \end{array}$$

Table 1 provides variable definitions and descriptive statistics. After obtaining the fitted values from Equation 2, we match, without replacement, each client of a Big 4 auditor with a client of a non-Big 4 auditor that has the closest fitted value in the same fiscal year and corresponding two-digit SIC (Standard Industrial Classification) code industry within a maximum distance of 0.03 between the two propensity scores. This procedure creates a pseudo-random sample in which one group of firms (the treatment group) is audited by Big 4 audit firms, while the other group (the control group) is not audited by Big 4 audit firms. As the variation in the client characteristics is minimized through the PSM procedure, the remaining differences in means between the treatment and control groups are justifiably considered the treatment effect.

To test the second set of hypotheses, we introduce the following two variables to investigate the incremental effect of Big 4 auditor choice on 10-K disclosure volume: (a) the magnitude of discretionary accruals and (b) the effective bid–ask spread. First, with regard to the measurement of opportunistic behavior, we estimate normal levels of accruals based on the modified Jones model^8^ (Dechow et al., 1995), which defines the accrual process as a function of growth in credit sales and investment in Property, plant & equipment (PPE), controlling for firm performance (Kothari et al., 2005). We then decompose total accruals into discretionary and non-discretionary components, with a larger magnitude of discretionary accruals (*ADA_MJR*) indicating more aggressive opportunistic behavior. Second, following the same approach as Hendershott et al. (2011), we measure the effective spread (*EFFSPRD*) as the difference between the bid–ask midpoint and the actual transaction price divided by the bid–ask midpoint. Specifically, we calculate a volume-weighted average over the 12-month period, with a larger effective spread indicating less stock liquidity and, hence, more information asymmetry.

Because we expect that the benefit of enhanced disclosures provided by Big 4 auditors will be more pronounced for audit clients with poorer accrual quality and for those with higher information asymmetry, we partition the sample into two subsamples using the median of *ADA_MJR* and *EFFSPRD* to examine the differential effects of Big 4 auditor choice together with its incremental effect through an interaction between *BIG4* and either *ADA_MJR* or *EFFSPRD* in the disclosure model.

To test the last set of hypotheses, we follow the methodology introduced by Hribar et al. (2014) and obtain a measure of residual disclosure (*RES_WRD*) as the portion of 10-K disclosure volume unexplained by observable client characteristics and operating complexity (e.g., Cazier & Pfeiffer, 2015; Li, 2008). In particular, we regress the natural logarithm of 10-K disclosure volume on a set of explanatory variables (Equation 1), excluding an indicator variable of Big 4 firms, in the disclosure model by fiscal year. By construction, this measurement choice constrains mean residual disclosure to be equal to zero. Firms with positive (negative) residuals can be interpreted as having abnormally long (short) 10-K disclosures.

Building on prior studies, we then investigate whether abnormally long disclosures trigger a variety of auditor responses through additional audit effort, as evidenced by either higher audit fees or longer audit report lags. Specifically, we estimate Equation 3 (the audit fee model) and Equation 4 (the audit report lag model) with the inclusion of control variables based on audit fee studies (e.g., Hay, 2013; Hay et al., 2006; Simunic, 1980) and audit report lag studies (e.g., Amin et al., 2018; Knechel & Payne, 2001), as described in Table 1.

(3) $$\;\;\begin{array}{c} \mathrm{LNAFEE}S_{t}=\alpha_{0}+\alpha_{1}\mathrm{RES}\_\_\mathrm{WR}D_{t}+\alpha_{2}\mathrm{BIG}4_{t}+\alpha_{3}\mathrm{LNASSE}T_{t}\\ +\alpha_{4}\mathrm{CURREN}T_{t}+\alpha_{5}\mathrm{INVRE}C_{t}+\alpha_{6}\mathrm{LEVERAG}E_{t}+\alpha_{7}\mathrm{RO}A_{t}\\ +\alpha_{8}\mathrm{INT}L_{t}+\alpha_{9}MA_{t}+\alpha_{10}\mathrm{SPI}\_\_DM_{t}+\alpha_{11}\mathrm{LNBUSSE}G_{t}\\ +\alpha_{12}\mathrm{LOS}S_{t}+\alpha_{13}\mathrm{MT}B_{t}+\alpha_{14}\mathrm{BUS}Y_{t}+\alpha_{15}\mathrm{TENUR}E_{t}\\ +\alpha_{16}\mathrm{IP}O_{t}+\alpha_{17}\mathrm{SE}O_{t}+\alpha_{18}\mathrm{OPINIO}N_{t}+\alpha_{19}\mathrm{HIGHLI}T_{t}\\ +\mathrm{Year}\;\mathrm{and}\;\mathrm{Industry}\;\mathrm{Fixed}\;\mathrm{Effects}+\varepsilon_{t}, \end{array}$$

(4) $$\begin{array}{c} \mathrm{ADREPLA}G_{t}=\alpha_{0}+\alpha_{1}\mathrm{RE}S_{\mathrm{WR}D_{t}}+\alpha_{2}\mathrm{SIZ}E_{t}+\alpha_{3}\mathrm{LR}{G_{\mathrm{ACCEL}}}_{t}\\ +\alpha_{4}\mathrm{BIG}4_{t}+\alpha_{5}\mathrm{BUS}Y_{t}+\alpha_{6}CG_{t}+\alpha_{7}\mathrm{INT}L_{t}+\alpha_{8}\mathrm{LOS}S_{t}\\ +\alpha_{8}\mathrm{SPI}\_\_DM_{t}+\alpha_{9}\mathrm{ALTMA}N_{t}+\\ \mathrm{Year}\;\mathrm{and}\;\mathrm{Industry}\;\mathrm{Fixed}\;\mathrm{Effects}+\varepsilon_{\mathrm{it}}. \end{array}$$

### Sample Selection and Descriptive Statistics

#### Sample selection

We obtain the available datasets from Loughran and McDonald (2014) and focus on the textual characteristics of 10-K annual reports available on EDGAR during the 2004–2014 period. These datasets contain various complexity and readability measures, including the word counts of the 10-K reports based on words appearing in the Loughran–McDonald Master Dictionary.

To address our research questions, we merge the datasets with Compustat fundamental annual files and CRSP monthly stock files to obtain the necessary financial statement data for all firm–years from 2004 to 2014. We exclude all observations related to financial (between SIC 6000 and 6999) and utility (between SIC 4900 and 4949) firms. We delete firms with total assets of less than $1 million and negative book value of equity, as well as firms that have fewer than 2,000 words in their 10-K reports. We also require that firms have a stock price of at least $1 or a total market capitalization greater than or equal to $200 million. After imposing all the necessary requirements to the estimated disclosure model, we obtain a sample of 43,575 firm–year observations, in which 13,818 (31.7%) and 29,757 (68.3%) reflect non-Big 4 and Big 4 accounting clients, respectively. Using Equation 2 to calculate the propensity scores and imposing a caliper distance of 3%, we obtain a PSM sample of 13,152 firm–years, of which 6,576 are Big 4 clients and 6,576 are non-Big 4 clients. Finally, we winsorize observations that fall in the top and bottom 1% of the distribution for each non-discrete variable to mitigate potential problems of outliers in both samples.

#### Descriptive statistics

Table 1 reports the descriptive statistics for all variables used in the disclosure model (Equation 1) during the 2004–2014 period. Panel A reports the mean summary statistics for the full sample of Big 4 and non-Big 4 auditors together with their differences in means. Overall, the descriptive results illustrate that clients of Big 4 auditors are relatively larger in size, more profitable, and have more leverage than those of non-Big 4 auditors. We also document that the mean *LNWORDS* of Big 4 and non-Big 4 clients are 10.80 and 10.45, which translates into means of 49,026 and 34,493 words, respectively, indicating that clients of large audit firms tend to provide more detailed disclosures than do those of small audit firms. In Panel B, the PSM sample based on the auditor selection model results in a total sample of 13,152 observations with relatively similar client characteristics in which one group of firms is audited by Big 4 and the other group is audited by non-Big 4 auditors.^9^ While the PSM model appears effective in forming a balanced sample of Big 4 and non-Big 4 auditors, we consistently find that the average 10-K disclosure volume is still relatively larger for clients of Big 4 auditors (10.61) than those of non-Big 4 auditors (10.57).

Table 2 reports the Pearson (above diagonal) and the Spearman (below diagonal) correlation coefficients among the key variables used in this study. First, the high correlation between *LNWORDS* and *SIZE* $\left(r_{p}=r_{s}=0.46\right)$ is consistent with prior studies, suggesting that a significant portion of 10-K length is attributable to operating complexity. We also find that *BIG4* is positively correlated with *LNWORDS* $\left(r_{p}=r_{s}=0.32\right)$ and *RES_WRD* $\left(r_{p}=0.04,\;r_{s}=0.05;\;p<.01\right)$ , indicating that the influence of Big 4 auditors potentially contributes to the variation in 10-K disclosure volume. As expected, the significant correlations between *RES_WRD* and both *LNAFEES* $\left(r_{p}=0.10,\;r_{s}=0.11\right)$ and *ADREPLAG* $\left(r_{p}=0.09,\;r_{s}=0.08\right)$ indicate that abnormally long disclosures are associated with higher audit fees and longer audit report lags (*p* < .01 for both).

**Table 2.** Correlations.

Variable		[1]	[2]	[3]	[4]	[5]	[6]	[7]	[8]
[1]	*LNWORDS*	1.00	**.74**	**.32**	**.46**	**–.08**	**–.26**	**.54**	**–.10**
			43,575	43,575	43,575	34,573	40,262	43,341	43,575
[2]	*RES_WRD*	**.70**	1.00	**.05**	.00	**.02**	**–.03**	**.11**	**.09**
		43,575		43,575	43,575	34,573	40,262	43,341	43,575
[3]	*BIG4*	**.32**	**.04**	1.00	**.58**	**–.19**	**–.44**	**.63**	**–.26**
		43,575	43,575		43,575	34,573	40,262	43,341	43,575
[4]	*SIZE*	**.46**	0.00	**.59**	1.00	**–.22**	**–.60**	**.81**	**–.46**
		43,575	43,575	43,575		34,573	40,262	43,341	43,575
[5]	*ADA_MJR*	**–.09**	**.02**	**–.17**	**–.22**	1.00	**.13**	**–.22**	**.16**
		34,573	34,573	34,573	34,573		32,126	34,376	34,573
[6]	*EFFSPRD*	**–.37**	.00	**–.54**	**–.89**	**.19**	1.00	**–.49**	**.33**
		40,262	40,262	40,262	40,262	32,126		40,064	40,262
[7]	*LNAFEES*	**.53**	**.10**	**.64**	**.80**	**–.20**	**–.72**	1.00	**–.29**
		43,341	43,341	43,341	43,341	34,376	40,064		43,341
[8]	*ADREPLAG*	**–.19**	**.08**	**–.34**	**–.58**	**.18**	**.53**	**–.42**	1.00
		43,575	43,575	43,575	43,575	34,573	40,262	43,341	

*Note.* This table reports the Pearson (above diagonal) and the Spearman (below diagonal) correlation coefficients. Bold values are significant at .01 levels (two-tailed *p* values). *RES_WRD* = residual from estimating Equation 1 with the word count in the 10-K complete submission text file as the dependent variable. Other variables are defined in Table 1.

## Main Results

Table 3 reports the regression results of estimating the disclosure model with the inclusion of *BIG4* on both the full sample (Column 1) and the PSM sample (Column 2). While all explanatory variable coefficients are significant and have directional effects consistent with those documented in previous studies, we consistently find that the estimated coefficient of *BIG4* is positive and significant (coefficient = 0.07 with *t*-statistic = 7.64 for the full sample; coefficient = 0.04 with *t*-statistic = 4.09 for the PSM sample), indicating that the variation in 10-K reports between Big 4 and non-Big 4 auditors persists with the PSM sample.^10^ This result suggests that the clients of Big 4 auditors benefit from improved disclosure quality through longer and more detailed 10-K reports. It is important to address the economic significance of the results. Specifically, the findings suggest that the choice of Big 4 auditors is associated with a 7.11% increase in the number of words contained in 10-K reports, which translates into approximately 3,120 words. Thus, the clients of Big 4 auditors benefit from improved disclosure quality through longer and more detailed 10-K reports.^11^

**Table 3.** Auditor Choice and 10-K Disclosure Volume.

Variables	(1)		(2)	
	Full sample		PSM sample	
	DV = *LNWORDS*		DV = *LNWORDS*	
	Coefficient	*t*-statistic	Coefficient	*t*-statistic
Intercept	9.80***	67.38	10.20***	47.63
***BIG4***	**0.07** ***	**7.64**	**0.04** ***	**4.09**
*DELTA_ROA*	0.00**	–2.47	0.00	0.17
*DELTA_REV*	0.01	1.23	0.00	0.36
*MA*	0.03***	5.21	0.02*	1.82
*FY_RET*	–0.04***	–14.69	–0.05***	–9.46
*SD_RETURN*	0.96***	18.57	1.00***	13.49
*SPI_DM*	0.10***	18.99	0.10***	12.58
*CAP_LEASE*	0.06***	7.27	0.06***	4.35
*OP_LEASE*	0.02*	1.92	0.01	0.98
*RD*	0.32***	6.89	0.20***	2.94
*INTANG*	–0.03	–1.58	0.01	0.23
*SIZE*	0.12***	43.50	0.12***	25.63
*AGE*	–0.07***	–17.48	–0.08***	–13.10
*MTB*	–0.05***	–19.69	–0.05***	–10.86
*LEVERAGE*	0.31***	14.36	0.37***	11.62
*FCF*	–0.42***	–14.81	–0.40***	–10.09
*DERIVATIVE*	0.04***	5.79	0.05***	3.89
*LNBUSSEG*	0.05***	6.25	0.07***	5.13
*LNGEOSEG*	0.00	0.37	0.01	1.32
*SD_OIADP*	0.03**	2.30	0.03**	2.00
*DELAWARE*	0.04***	4.34	0.04***	3.71
*IPO*	0.10*	1.65	0.13	1.10
*SEO*	0.04***	4.35	0.02	1.55
*NMCOUNT*	0.03	1.11	–0.03	–0.91
Fixed effects	Yes		Yes	
Observations	43,575		13,152	
Adjusted *R*^2^	42.9%		37.7%	

*Note.* This table reports the regression results of estimating the disclosure model (Equation 1) on both the full sample (Column 1) and the PSM sample (Column 2). The *t*-statistic is determined by clustered standard errors at firm level. PSM = propensity score matching. Bold values are significant at .01 levels (two-tailed p values).

* , **, and *** denote significance at the .10, .05, and .01 levels, respectively.

To examine the incremental effect of Big 4 auditors on 10-K length in situations where financial reporting users potentially need more information to understand the effects of material transactions or events reported in the financial disclosure, we estimate the disclosure model (Equation 1) with the inclusion of either *ADA_MJR* or *EFFSPRD* and its interaction with *BIG4* in Table 4. We then partition the full sample into subsamples with low and high values of *ADA_MJR* in Columns (1) and (2), and subsamples with low and high values of *EFFSPRD* in Columns (3) and (4), respectively.

**Table 4.** Incremental Effect of Big-4 Auditors on 10-K Disclosure Volume.

Variables	(1)		(2)		(3)		(4)	
	Low *ADA_MJR*		High *ADA_MJR*		Low *EFFSPRD*		High *EFFSPRD*	
	DV = *LNWORDS*		DV = *LNWORDS*		DV = *LNWORDS*		DV = *LNWORDS*	
	Coefficient	*t*-statistic	Coefficient	*t*-statistic	Coefficient	*t*-statistic	Coefficient	*t*-statistic
Panel A: The full sample								
*BIG4*	0.06***	3.44	0.02	1.52	–0.03	–0.88	0.07***	6.19
*ADA_MJR*	–0.30	–0.55	–0.05	–0.66				
*ADA_MJR*×*BIG4*	0.28	0.44	0.34***	3.20				
*EFFSPRD*					–0.36**	–2.13	–0.02***	–4.64
*EFFSPRD*×*BIG4*					0.29*	1.72	0.00	–0.29
Control variables	Yes		Yes		Yes		Yes	
Observations	17,103		17,470		20,128		20,134	
Adjusted *R*^2^	43.1%		43.9%		31.8%		38.5%	
Panel B: The PSM sample								
*BIG4*	0.04*	1.74	–0.01	–0.39	0.01	0.20	0.05***	3.84
*ADA_MJR*	–0.33	–0.49	–0.06	–0.58				
*ADA_MJR*×*BIG4*	0.19	0.20	0.30*	1.88				
*EFFSPRD*					0.01	0.07	–0.03***	–3.98
*EFFSPRD*×*BIG4*					0.00	–0.02	–0.01	–0.75
Control variables	Yes		Yes		Yes		Yes	
Observations	4,573		5,695		2,873		9,309	
Adjusted *R*^2^	39.1%		38.1%		34.0%		35.8%	

*Note.* This table reports the benefit of enhanced disclosures provided by Big 4 auditors for audit clients with poorer accrual quality and those with higher information asymmetry. The full sample (Panel A) and the PSM sample (Panel B) are partitioned into subsamples with low and high values of *ADA_MJR* in Columns (1) and (2), and subsamples with low and high values of *EFFSPRD* in Columns (3) and (4), respectively. The *t*-statistic is determined by clustered standard errors at firm level. PSM = propensity score matching.

* , **, and *** denote significance at the .10, .05, and .01 levels, respectively.

As expected, we find that the coefficient of *BIG4* is positive and significant (coefficient = 0.06 with *t*-statistic = 3.44 for the full sample; coefficient = 0.04 with *t*-statistic = 1.74 for the PSM sample) in the subsample of firms with better accrual quality. Similarly, in the subsample of firms with poorer accrual quality, we find that the incremental effect of Big 4 auditors, as captured by the estimated coefficient of *ADA_MJR*×*BIG4* (coefficient = 0.34 with *t*-statistic = 3.20 for the full sample; coefficient = 0.30 with *t*-statistic = 1.88 for the PSM sample), is relatively larger than those reported in the first column. Alternatively, while we find marginal results or no relation in the subsample of firms with lower levels of information asymmetry, the estimated coefficient of *BIG4* is positive and significant in the subsample of firms with higher levels of information asymmetry (coefficient = 0.07 with *t*-statistic = 6.19 for the full sample; coefficient = 0.05 with *t*-statistic = 3.84 for the PSM sample). Overall, these results provide evidence supporting an auditor influence in increasing the informativeness of client disclosures, particularly when audit clients report higher levels of discretionary accruals or experience higher levels of information asymmetry.

Next, we estimate the disclosure model by year and obtain residual disclosures as the portion of 10-K disclosure volume unexplained by observable client characteristics and operating complexity. *RES_WRD* is defined as residuals from estimating the disclosure model using the word count (*LNWORDS*) of the complete 10-K submission text file as the dependent variable. Specifically, we investigate whether 10-K disclosure volume varies with the auditor’s influence and induces higher audit effort through charging a fee premium or longer audit report lags. The descriptive statistics for all the variables (and their definitions) used in both models are reported in Table 5.

**Table 5.** Descriptive Statistics—The Audit Fee and Audit Report Lag Models.

Variables	*n*	*M*	*SD*	Median	Q1	Q3
Panel A: Variables used in the audit fee model						
*LNAFEES*	30,918	13.68	1.26	13.71	12.85	14.51
*BIG4*	30,918	0.72	0.45	1.00	0.00	1.00
*LNASSET*	30,918	6.12	2.04	6.08	4.65	7.51
*CURRENT*	30,918	3.09	3.32	2.15	1.43	3.47
*INVREC*	30,918	0.26	0.19	0.23	0.11	0.37
*LEVERAGE*	30,918	0.18	0.19	0.14	0.00	0.30
*ROA*	30,918	0.00	0.18	0.04	–0.02	0.08
*INTL*	30,918	0.08	0.28	0.00	0.00	0.00
*MA*	30,918	0.20	0.40	0.00	0.00	0.00
*SPI_DM*	30,918	0.67	0.47	1.00	0.00	1.00
*LNBUSSEG*	30,918	1.00	0.51	0.69	0.69	1.39
*LOSS*	30,918	0.31	0.46	0.00	0.00	1.00
*MTB*	30,918	2.03	1.48	1.55	1.16	2.31
*BUSY*	30,918	0.68	0.47	1.00	0.00	1.00
*TENURE*	30,918	9.37	8.62	7.00	3.00	13.00
*IPO*	30,918	0.00	0.00	0.00	0.00	0.00
*SEO*	30,918	0.07	0.26	0.00	0.00	0.00
*OPINION*	30,918	0.33	0.47	0.00	0.00	1.00
*HIGHLIT*	30,918	0.36	0.48	0.00	0.00	1.00
Panel B: Variables used in the audit report lag model						
*ADREPLAG*	43,575	4.16	0.25	4.16	4.03	4.30
*RES_WRD*	43,575	0.00	0.37	–0.04	–0.25	0.20
*SIZE*	43,575	6.17	2.01	6.13	4.67	7.56
*LRG_ACCEL*	43,575	0.35	0.48	0.00	0.00	1.00
*BIG4*	43,575	0.68	0.47	1.00	0.00	1.00
*BUSY*	43,575	0.74	0.44	1.00	0.00	1.00
*GC*	43,575	0.02	0.12	0.00	0.00	0.00
*INTL*	43,575	0.07	0.26	0.00	0.00	0.00
*LOSS*	43,575	0.26	0.44	0.00	0.00	1.00
*SPI_DM*	43,575	0.61	0.49	1.00	0.00	1.00
*ALTMAN*	43,575	3.84	6.20	2.47	0.77	4.76

*Note.* This table reports the summary statistics of variables used for the audit fee model (Panel A) and the audit report lag model (Panel B) from fiscal year 2004 to 2014. All continuous variables are winsorized at the 1st and 99th percentiles. Variable definitions for audit fee model: *LNAFEES* = the natural logarithm of audit fees; *BIG4* = indicator variable equal to one if the firm’s auditor is a member of the Big 4 audit firms (PwC, EY, KPMG, and Deloitte), and zero otherwise; *LNASSET* = the natural logarithm of total assets (in millions); *CURRENT* = the ratio of current assets to current liabilities; *INVREC* = the ratio of total inventory and receivables to total assets; *LEVERAGE* = the sum of short-term and long-term debt, divided by total assets; *ROA* = income before extraordinary items, scaled by average total assets; *INTL* = indicator variable equal to one if an audit’s client has international operations, and zero otherwise; *MA* = indicator variable equal to one if an audit’s client is engaged in a merger or acquisition (as reported by SDC Platinum) during the year, and zero otherwise; *SPI_DM* = indicator variable equal to one if an audit’s client has a special item during the year, and zero otherwise; *LNBUSSEG* = the natural logarithm of one plus the number of business segments; *LOSS* = indicator variable equal to one if income before extraordinary items is negative in the current period, and zero otherwise; *MTB* = the firm’s market value divided by its book value; *BUSY* = indicator variable equal to one if an audit’s client has a year-end fall on December 31, and zero otherwise; *TENURE* = the number of years the company has been audited by the same audit firm; *IPO* = indicator variable equal to one if an audit’s client is engaged in an initial public offering during the year, and zero otherwise; *SEO* = indicator variable equal to one if an audit’s client is engaged in a seasoned equity offering (as reported by SDC Platinum) during the year, and zero otherwise; *OPINION* = indicator variable equal to one if an audit’s client receives a modified audit opinion, and zero otherwise, where a modified opinion is defined as anything except a standard unqualified audit opinion coded as one by Compustat; *HIGHLIT* = indicator variable equal to one for high litigation risk industries as defined in J. Francis et al. (1994), and zero otherwise. Variable definitions for audit report lag model: *ADREPLAG* = the natural logarithm of the number of days from the fiscal year-end to the audit report date; *RES_WRD* = the residual from estimating the disclosure model using the word count of the complete 10-K submission text file as the dependent variable; *SIZE* = the natural logarithm of the firm’s market value at the end of the fiscal year; *LRG_ACCEL* = indicator variable equal to one if an audit’s client is a large accelerated filer, and zero otherwise; *BIG4* = indicator variable equal to one if the firm’s auditor is a member of the Big 4 (PwC, EY, KPMG, and Deloitte), and zero otherwise; *BUSY* = indicator variable equal to one if an audit’s client has a year-end fall on December 31, and zero otherwise; *GC* = indicator variable equal to one if a firm receives a going-concern report in a fiscal period, and zero otherwise; *INTL* = indicator variable equal to one if an audit’s client has international operations, and zero otherwise; *LOSS* = indicator variable equal to one if income before extraordinary items is negative in the current period, and zero otherwise; *SPI_DM* = indicator variable equal to one if an audit’s client has a special item during the year, and zero otherwise; *ALTMAN* = the Altman *z*-score.

**Figure 1.**
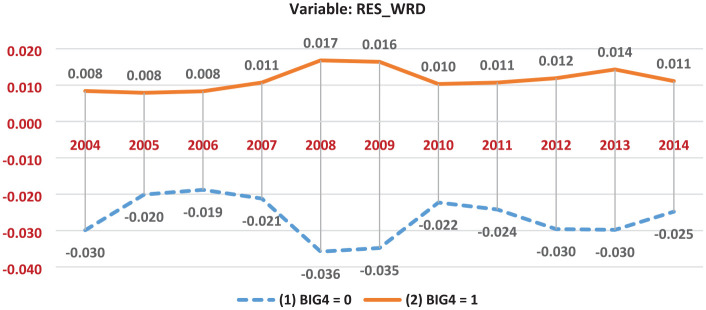
Residual disclosure of the 10-K reports. *Note.* Figure 1 depicts the mean residual disclosure of firms that use Big 4 auditors (*BIG4* = 1) and non-Big 4 auditors (*BIG4* = 0) by fiscal year.

Furthermore, Figure 1 depicts the mean residual disclosure of firms that use Big 4 auditors (*BIG4* = 1) and non-Big 4 auditors (*BIG4* = 0) over time. As illustrated in Figure 1, the mean *RES_WRD* values of firms that use Big 4 (non-Big 4) auditors are consistently positive (negative) and do not fluctuate around zero throughout the sample period.

We estimate the audit fee model (Equation 3) and report the regression results in Table 6.^12^ Consistent with our prediction, we find that the estimated coefficient of *RES_WRD* is positive and significant (*p* < .01; see Column 1). We also partition the full sample into subsamples of non-Big 4 clients (Column 2) and Big 4 clients (Column 3). The results are consistent with abnormally long disclosures containing information on unobserved audit costs in response to increased auditor effort across both subsamples (coefficient = 0.24 with *t*-statistic = 9.75 for the subsample of non-Big 4 clients; coefficient = 0.18 with *t*-statistic = 11.52 for the subsample of Big 4 clients). In terms of economic significance, a one-standard-deviation increase in residual disclosures leads to an 8.04% (or about $7,439) increase in audit fees.

**Table 6.** Residual Disclosures and Audit Fees (Level Specification).

Variables	(1)		(2)		(3)	
	Full sample		Non-Big 4 firms		Big 4 firms	
	DV = *LNAFEES*		DV = *LNAFEES*		DV = *LNAFEES*	
	Coefficient	*t*-statistic	Coefficient	*t*-statistic	Coefficient	*t*-statistic
Intercept	10.05***	113.77	10.16***	71.18	10.40***	90.01
*RES_WRD*	0.21***	15.14	0.24***	9.75	0.18***	11.52
*LNASSET*	0.52***	98.05	0.55***	51.59	0.51***	81.23
*CURRENT*	–0.02***	–9.74	–0.02***	–7.89	–0.02***	–6.68
*INVREC*	0.56***	11.16	0.29***	4.38	0.76***	11.30
*LEVERAGE*	–0.25***	–6.43	–0.36***	–5.72	–0.17***	–3.77
*ROA*	–0.39***	–10.26	–0.26***	–4.85	–0.47***	–9.48
*INTL*	0.12***	4.43	0.04	0.60	0.16***	5.28
*MA*	0.02***	2.65	0.02	0.91	0.03***	2.62
*SPI_DM*	0.17***	17.05	0.13***	8.83	0.17***	14.04
*LNBUSSEG*	0.05***	4.18	0.05*	1.71	0.05***	3.86
*LOSS*	0.10***	7.60	0.12***	5.57	0.08***	5.58
*MTB*	0.01**	2.34	0.02***	3.10	0.01	1.44
*BUSY*	0.05***	3.12	0.04	1.64	0.05**	2.46
*TENURE*	0.00	–0.71	–0.01***	–2.91	0.00	0.75
*SEO*	0.04***	2.65	0.00	–0.09	0.04***	2.78
*OPINION*	0.12***	12.16	0.16***	8.63	0.10***	9.36
*HIGHLIT*	0.04	1.48	0.09**	2.41	0.02	0.76
*BIG4*	0.40***	22.20				
Fixed effects	Yes		Yes		Yes	
Observations	30,918		8,627		22,291	
Adjusted *R*^2^	84.4%		71.8%		77.9%	

*Note.* This table presents regression results of the audit fee model (Equation 3) for the full sample and for subsamples partitioned on Big 4 and non-Big 4 firms. The *t*-statistic is determined by clustered standard errors at firm level.

* , **, and *** denote significance at the .10, .05, and .01 levels, respectively, using two-tailed tests.

Furthermore, we use the change specification to examine the relationship between residual disclosures and the level of audit fees. The change variables in the model (denoted by Δ) are then measured as the current year value, less the prior year value, of the variables used in the audit fee model. As expected, we find that the year-to-year change in the residual disclosures (*ΔRES_WRD*) is positively associated with the year-to-year change in the level of audit fees (coefficient = 0.03 with *t*-statistic = 5.82). This result suggests that firms with an unexpected increase in 10-K disclosure volume pay higher audit fees on average than in the immediately preceding year. In addition, we partition the full sample into subsamples of non-Big 4 clients (Column 2) and Big 4 clients (Column 3), and the results consistently show that the estimated coefficients on *ΔRES_WRD* are positive and significant across both subsamples (coefficient = 0.03 with *t*-statistic = 1.89 for the subsample of non-Big 4 auditors; coefficient = 0.03 with *t*-statistic = 5.57 for the subsample of Big 4 auditors; Table 7).

**Table 7.** Residual Disclosures and Audit Fees (Change Specification).

Variables	(1) Full sample		(2) Non-Big 4 firms		(3) Big 4 firms	
	DV = ΔLNAFEES		DV = *ΔLNAFEES*		DV = *ΔLNAFEES*	
	Coefficient	*t*-statistic	Coefficient	*t*-statistic	Coefficient	*t*-statistic
Intercept	0.05	1.60	0.02	0.27	0.06*	1.91
*Δ* *RES_WRD*	0.03***	5.82	0.03*	1.89	0.03***	5.57
*ΔLNASSET*	0.32***	27.48	0.31***	14.38	0.33***	23.94
*ΔCURRENT*	–0.01***	–5.05	0.00*	–1.70	–0.01***	–5.67
*ΔINVREC*	0.12***	3.00	0.10	1.55	0.12**	2.32
*ΔLEVERAGE*	0.05*	1.70	–0.01	–0.24	0.08**	2.45
*ΔROA*	–0.23***	–10.66	–0.28***	–7.10	–0.22***	–8.16
*ΔLNBUSSEG*	0.01	1.17	0.02	0.96	0.00	0.33
*ΔMTB*	0.00	–0.82	–0.01**	–2.08	0.00	0.99
*ΔMA* (0 to 1)	0.02***	3.96	0.02*	1.74	0.02***	3.07
*ΔMA* (1 to 0)	–0.01	–1.23	–0.01	–0.38	–0.01	–1.47
*ΔSPI_DM* (0 to 1)	0.03***	4.93	0.04***	3.41	0.03***	4.37
*ΔSPI_DM* (1 to 0)	–0.02***	–4.20	–0.02*	–1.74	–0.02***	–3.50
*ΔOPINION* (0 to 1)	0.04***	4.47	0.05***	3.03	0.03***	3.60
*ΔOPINION* (1 to 0)	0.00	–0.12	–0.03**	–2.44	0.00	0.03
*ΔLOSS* (0 to 1)	0.03***	4.16	0.00	–0.01	0.05***	4.85
*ΔLOSS* (1 to 0)	0.00	–0.02	–0.01	–0.77	0.00	0.44
*ΔIPO* (1 to 0)	–0.08	–1.15	–0.18*	–1.95	0.03	0.35
*ΔSEO* (0 to 1)	0.03***	2.94	0.03	1.35	0.03***	2.66
*ΔSEO* (1 to 0)	–0.05***	–6.03	–0.04*	–1.85	–0.05***	–5.51
*ΔBIG4* (0 to 1)	0.30***	6.34				
*ΔBIG4* (1 to 0)	–0.36***	–16.15				
Fixed effects	Yes		Yes		Yes	
Observations	24,894		6,708		18,186	
Adjusted *R*^2^	16.5%		11.5%		15.2%	

*Note.* This table presents regression results of the audit fee model (Equation 3) using the change specification. The dependent variable is a year-to-year change in the level of audit fees (*ΔLNAFEES*). The *t*-statistic is determined by clustered standard errors at firm level.

* , **, and *** denote significance at the .10, .05, and .01 levels, respectively, using two-tailed tests.

Finally, we report the estimation results of the audit report lag model (Equation 4) in Table 8. The audit report lag (*ADREPLAG*) is defined as the period of time between the end of the fiscal year and the date that the audit report is signed. Consistent with our prediction, the estimated coefficient of *RES_WRD* is positive and significant (*p* < .01), indicating that the audit report lag is significantly associated with the unexplained portion of 10-K disclosure volume, which potentially captures the amount of time and effort that goes into completing financial statement audits. In terms of economic significance, a one-standard-deviation increase in residual disclosures translates to a 2.18% (or about 1.4 days) increase in audit report lag.

**Table 8.** Residual Disclosures and Audit Report Lags.

Variables	Full sample	
	DV = ADREPLAG	
	Coefficient	*t*-statistic
Intercept	4.46***	199.32
*RES_WRD*	0.06***	14.76
*SIZE*	–0.03***	–23.98
*LRG_ACCEL*	–0.13***	–29.01
*BIG4*	–0.00	–0.20
*BUSY*	0.02***	4.21
*GC*	0.12 ***	11.73
*INTL*	0.01*	1.67
*LOSS*	0.03***	7.81
*SPI_DM*	0.02***	6.32
*ALTMAN*	–0.00**	–2.35
Fixed effects	Yes	
Observations	43,575	
Adjusted *R*^2^	27.6%	

*Note.* This table presents regression results of the audit report lag model (Equation 4). The *t*-statistic is determined by clustered standard errors at firm level.

* , **, and *** denote significance at the .10, .05, and .01 levels, respectively, using two-tailed tests.

## Conclusion

In this study, we extend the literature by investigating whether the choice of Big 4 auditors contributes to cross-sectional variations in 10-K disclosure volume. In addition to the ample evidence that Big 4 auditors deliver higher assurance levels than non-Big 4 auditors, we document that Big 4 auditors also produce a higher quality of financial reporting such that audit clients improve the informativeness of their disclosures as measured by 10-K disclosure volume. We further show that this relation is more pronounced in situations where the users of financial reports potentially need more relevant information to understand the information conveyed in the 10-K reports. Together, these results suggest that audit services have at least two dimensions, *assurance level* and *financial reporting quality*, and that Big 4 audits are quality differentiated from non-Big 4 audits on both dimensions. Our work indirectly addresses the controversial issue regarding auditors’ responsibilities raised in the study by DeFond et al. (2016) and supports the broader view of auditors’ responsibilities, which argues that the role of the auditor is not limited to merely verifying GAAP compliance.

Because auditors are responsible for examining firms’ financial reporting and expressing an opinion on its fairness, we show that an abnormally high level of disclosure volume is positively associated with higher audit fees and longer audit report lags, thus indicating that a significant discretionary component of 10-K disclosure volume is associated with an increase in audit effort. As a result, researchers can use the size of the discretionary component of 10-K disclosures as another potentially useful proxy (besides audit hours and report lag) for audit effort. Overall, our findings show that auditors play more than a simple attestation role in the financial reporting process, and that the quality of financial reporting in a company’s 10-K annual report is a joint product of the effort and decisions of both a company’s managers and its auditors.

Finally, our research raises interesting questions about sources of demand for higher *financial reporting quality*, as well as characteristics of market equilibrium when audit services are differentiated on two dimensions. An audit firm’s decisions about service features and pricing are complex and need to consider not only what clients value and are willing to pay but also how competitors will react. For example, Vandenbosch and Weinberg (1995) analyze optimal product design and pricing strategies in a two-dimensional vertical differentiation model and find that, under certain conditions, maximum quality differentiation will occur on one product dimension with minimal differentiation on the other product dimension. Note that our empirical results are consistent with some level of quality differentiation between Big 4 and non-Big 4 firms on both service dimensions. A more detailed examination of these issues is left to future research. Examination of the factors that drive the demand for the higher *financial reporting quality* provided by Big 4 audits is also of interest. These factors may be the same as the sources of demand for higher *assurance*, such as the need for external financing, but other factors are also likely to be important. The results of our tests of Hypotheses 2a and 2b indicate that companies with poorer accrual quality and greater information asymmetry demand relatively higher quality financial reporting quality, but other factors may also enter into the demand for higher quality on this service dimension. Again, further examination of these issues is left to future research.

To conclude, we believe that modeling the audit service as having (at least) two dimensions—*assurance* and *financial reporting quality*—is useful in understanding the role of auditors in financial reporting and provides a new perspective on the nature of audit services.

## Supplemental Material

sj-pdf-1-jaf-10.1177_0148558X211062430 – Supplemental material for Auditor Choice and the Informativeness of 10-K Reports Supplemental material, sj-pdf-1-jaf-10.1177_0148558X211062430 for Auditor Choice and the Informativeness of 10-K Reports by Karel Hrazdil, Dan A. Simunic and Nattavut Suwanyangyuan in Journal of Accounting, Auditing & Finance

## Notes

1. The roundtable discussions on the evolving role of the auditor were sponsored by the Center of Audit Quality in 2011 and included the full range of financial reporting stakeholders—CEOs, CFOs, board and audit committee members, investors, auditors, former regulators, attorneys, and academics.

2. We believe that the basic notion that Big 4 audit firms provide differentially higher audit quality than non-Big 4 firms is not really in dispute. For example, an extensive analytical literature in auditing (e.g., Choi et al., 2008) posits a different loss function for Big 4 audit firms versus non-Big 4 firms, which then motivates Big 4 firms to deliver higher audit quality than non-Big 4 firms, ceteris paribus. Empirical studies cannot overturn this conceptualization of the problem because it is impossible to observe the counterfactuals (i.e., an audit of the same client at the same time performed by both a Big 4 firm and a non-Big 4 firm). Our results are, of course, consistent with this view of the world but represent only one of many ways that Big 4 audits can be quality differentiated from non-Big 4 audits.

3. Returning to the automobile example, a car that provides a high level of *style* could, at the same time, provide a high, equal, or low level of *safety*.

4. Mercer (2004) suggests that the credibility of financial reporting is influenced by various factors, including the degree of external assurance and the characteristics of the disclosure (e.g., precision, venue, horizon, and amount of supporting information). For example, supplementary disclosures are used as a firm strategy to enhance the credibility of earnings forecasts by increasing the content and *ex post* verifiability of them (e.g., Hutton et al., 2003).

5. Disclosure informativeness has been argued to be the main driver of AIMR scores (Lang & Lundholm, 1993) based on the assumption that financial analysts determine the disclosure quality of firms based on the adequacy of disclosure and the firm’s effectiveness in communicating with investors.

6. Lawrence et al. (2011) show that propensity score matching (PSM) on client characteristics eliminates the Big 4 effects and conclude that differences in quality between Big 4 and non-Big 4 audit firms largely reflect observable client characteristics, primarily client size.

7. While Shipman et al. (2017) state that the PSM results can be highly sensitive to design choices, such as caliper distance and whether matching was performed with or without replacement, DeFond et al. (2016) find that the results of Lawrence et al. (2011) arise in only a minority of design choices. In addition, DeFond et al. (2017) find evidence supporting the Big 4 effects for most of the audit quality measures in a majority of the matched samples.

8. The following accrual model is estimated with a minimum of 20 observations in each industry–year cluster:

$$TA_{t}=\alpha_{0}+\alpha_{1}\left(\frac{1}{AT_{t-1}}\right)+\alpha_{2}\left(\frac{\left(\Delta \mathrm{RE}V_{t}-\Delta \mathrm{RE}C_{t}\right)}{AT_{t-1}}\right)+\alpha_{3}\left(\frac{\mathrm{PP}E_{t}}{AT_{t-1}}\right)+\alpha_{4}\mathrm{RO}A_{t}+\varepsilon_{t},$$

where $TA_{t}$ = the difference between net income and operating cash flow in year *t*, scaled by lagged assets; $AT_{t-1}$ = lagged total assets; $\Delta \mathrm{RE}V_{t}-\Delta \mathrm{RE}C_{t}$ = the change in total revenues, less the change in total receivables in year *t* from year *t*– 1; $\mathrm{PP}E_{t}$ = the gross book value of property, plant, and equipment at the end of year *t*; and $\mathrm{RO}A_{t}$ = income before extraordinary items in year *t*, scaled by total assets.

9. The first-stage prediction used for estimating propensity scores in the auditors’ selection model is presented in the appendix (Supplementary Table). The pseudo-*R*^2^ and the area under the receiver operating characteristic (ROC) curve for *BIG4* are 58.8% and 0.91, respectively.

10. In untabulated results, there is no sign of a severe multicollinearity problem based on the variance inflation factor (VIF) values of each independent variable in the disclosure model. The inferences are unchanged when we implement the two-way clustering approach proposed by Petersen (2009), which is considered to be a conservative approach to control for time and firm effects in panel datasets.

11. After reviewing the DeFond et al. (2017) and Shipman et al. (2017) studies, we decided to use a two-stage Heckman selection model as an additional sensitivity test (details not tabulated). Consistent with the analyses using PSM, we find that the coefficient of *BIG4* is still positive and significant after controlling for the Mills variable. Alternatively, when we estimate the audit fee model for the subsample, where *BIG4* = 0 and *BIG4* = 1, the coefficient of *Mills_BIG4* is positive and significant in the subsample of Big 4 clients. Finally, to control for any year-specific events, such as the subprime financial crisis or the implementation of the Dodd–Frank Act, we estimate the disclosure model by year to allow the intercept and coefficients to vary by year. Untabulated results indicate that *BIG4* coefficients are significant and positively associated with 10-K length in every year of our sample period.

12. To prevent the model’s residuals from being correlated with client size, we estimate the audit fee model partitioned by asset size quintiles and find that the results are robust to client size subsample regressions.
