# Supplementary material for: Auditor Choice and the Informativeness of 10-K Reports
Source: J Account Audit Financ. 2021 Dec 15;39(2):388–413. doi: 10.1177/0148558X211062430 (PMC10913414; doi:10.1177/0148558X211062430)
Supplement: sj-pdf-1-jaf-10.1177_0148558X211062430 – Supplemental material for Auditor Choice and the Informativeness of 10-K Reports [file sj-pdf-1-jaf-10.1177_0148558X211062430.pdf]

Appendix: Supplementary Table

Table A1. First-stage prediction model

	(1) DV = BIG4	
Variables	Coef.	t-stat.
Intercept	–6.37	–0.71
LNASSET	0.60 ***	18.52
ATURN	0.09 ***	3.94
CURRENT	0.00	0.47
LEVERAGE	–0.31 ***	–2.96
ROA	–1.05 ***	–7.07
DELTA_ROA	0.00	–0.21
DELTA_REV	–0.31 ***	–8.98
MA	–0.10 **	–2.45
FY_RET	–0.16 ***	–7.08
SD_RETURN	–0.85 ***	–3.08
SPI_DM	0.16 ***	5.11
CAP_LEASE	–0.05	–1.36
OP_LEASE	0.35 ***	8.93
RD	3.36 ***	14.93
INTANG	–0.50 ***	–6.55
SIZE	0.62 ***	20.42
AGE	–0.02	–0.84
MTB	–0.06 ***	–3.80
FCFLOW	0.74 ***	3.92
DERIVATIVE	0.05	1.13
LNBUSSEG	–0.18 ***	–4.75
LNGEOSEG	0.12 ***	4.10
SD_OIADP	–0.30 ***	–4.76
DELAWARE	0.45 ***	14.11
IPO	–0.36	–0.92
SEO	–0.25 ***	–4.58
NMCOUNT	0.17	1.22
Fixed effects		Yes
Observations		43,575
Pseudo R-Squared		58.8%
Area under ROC curve		0.91

Note. This table presents the first-stage prediction used for estimating propensity scores in the auditors' selection model. *, **, *** denote significance at the 0.10, 0.05, and 0.01 levels, respectively.
